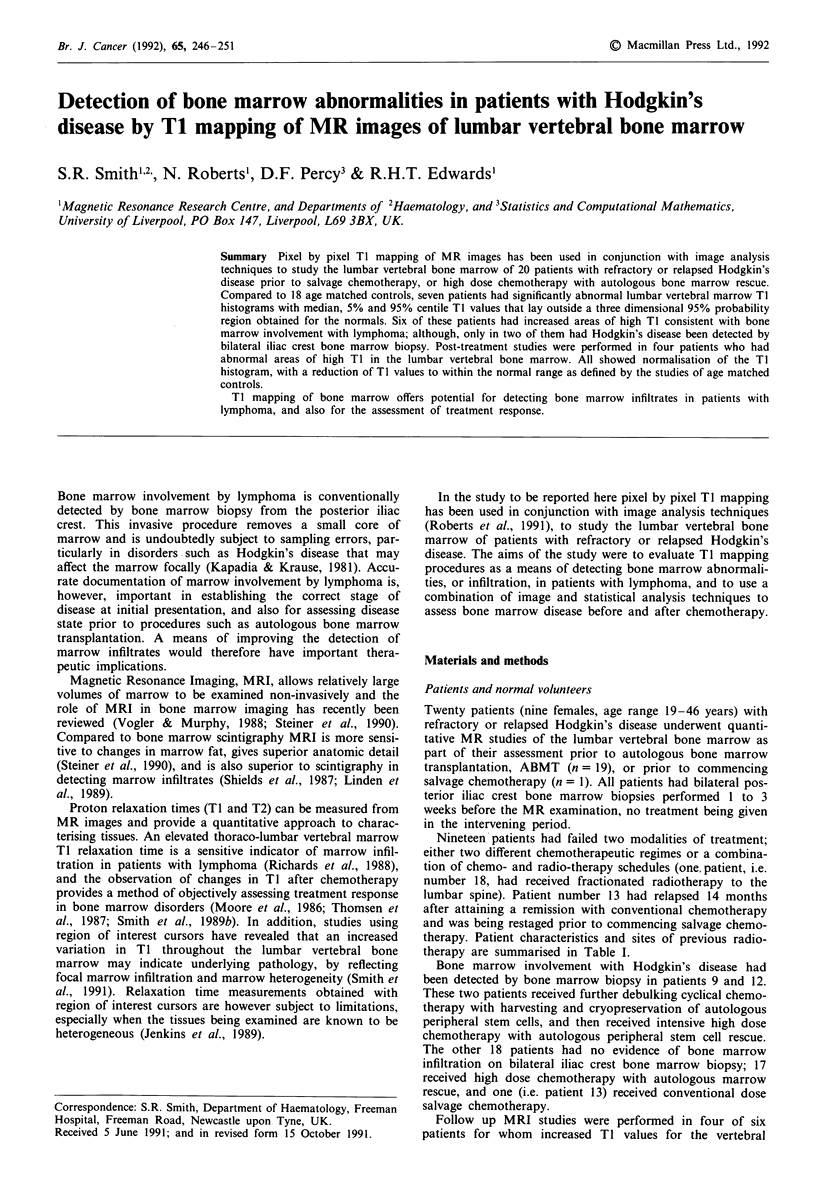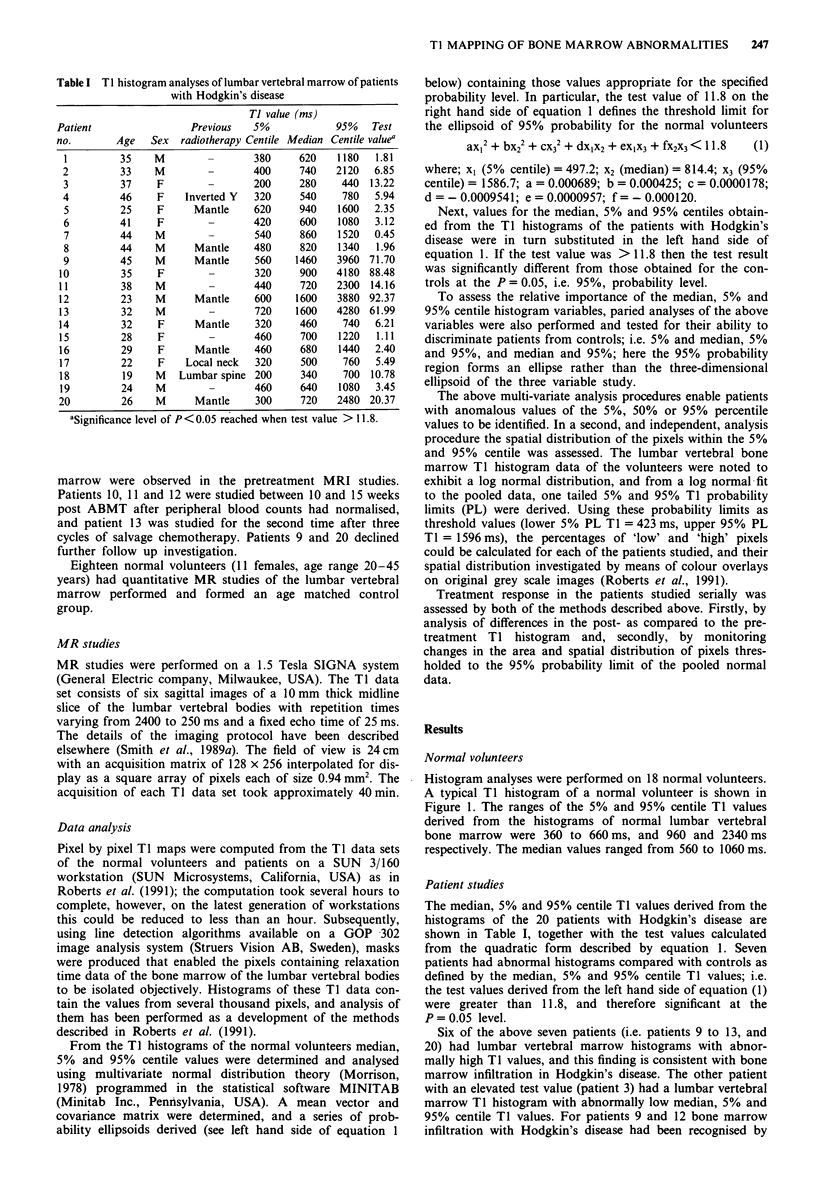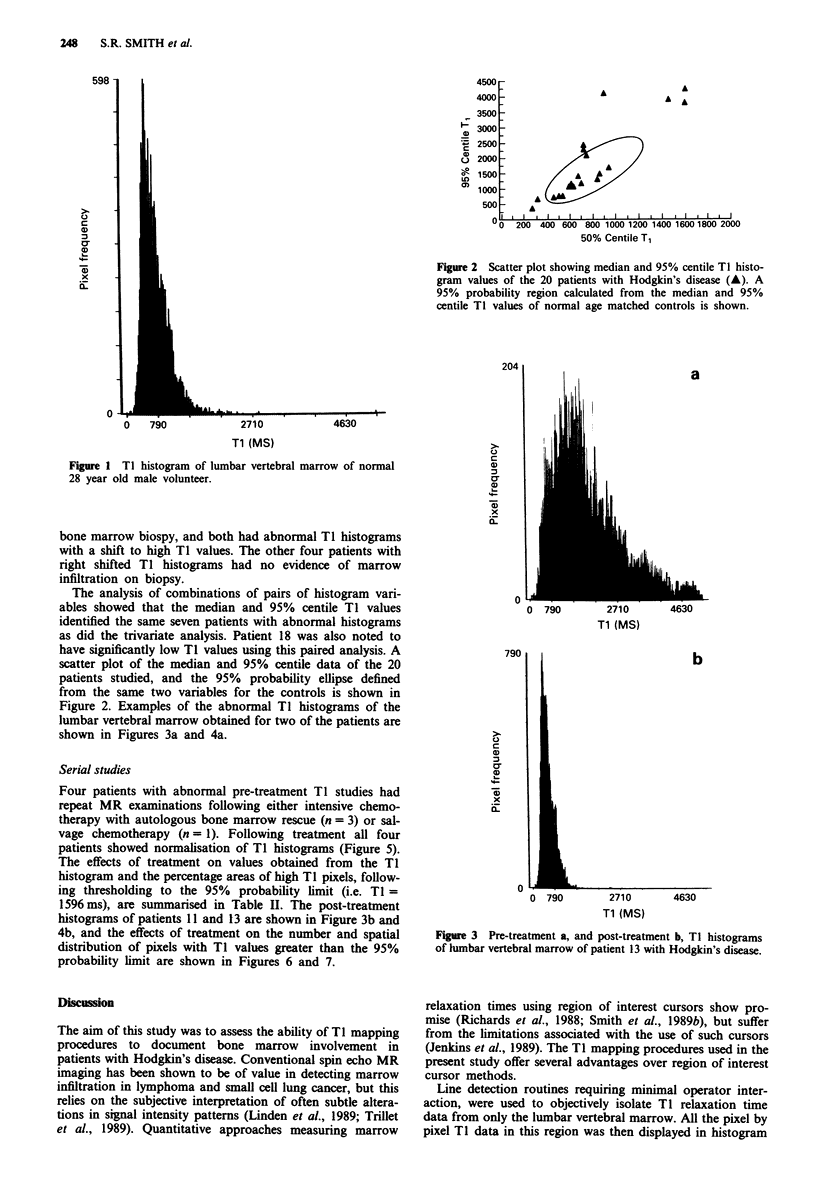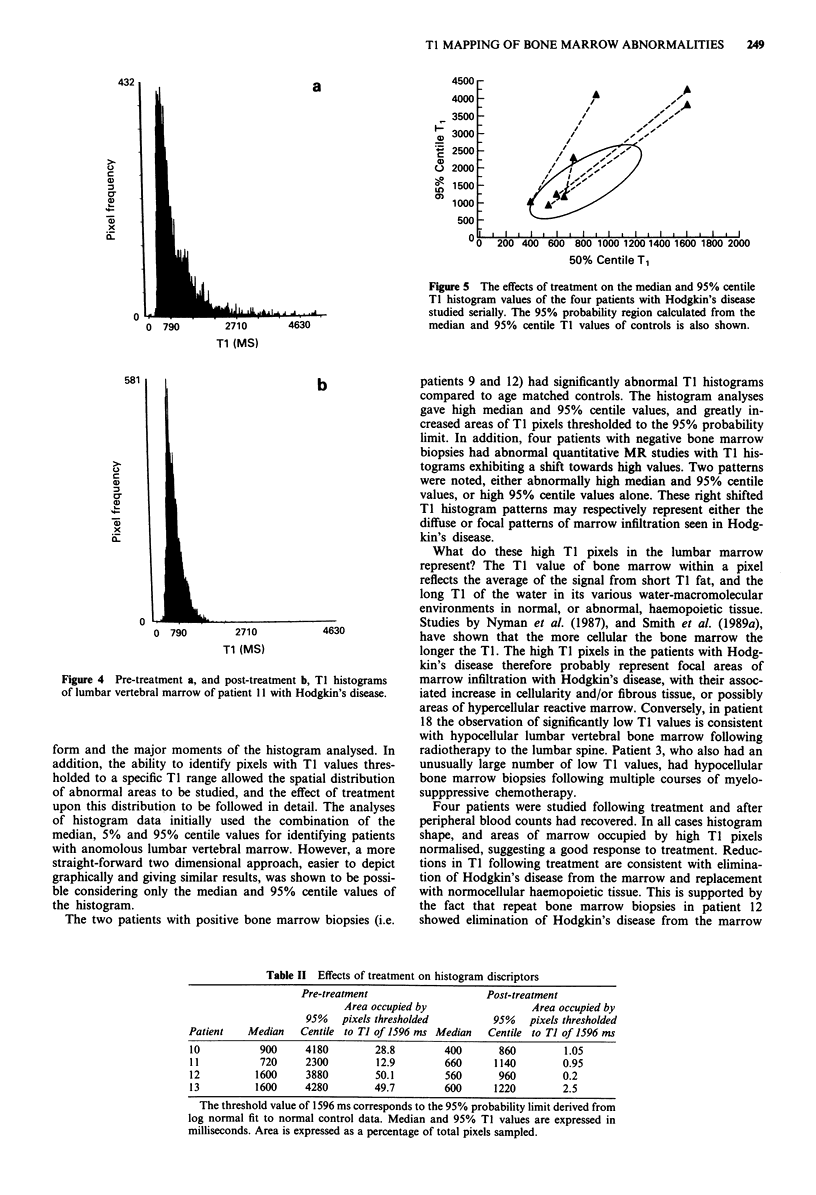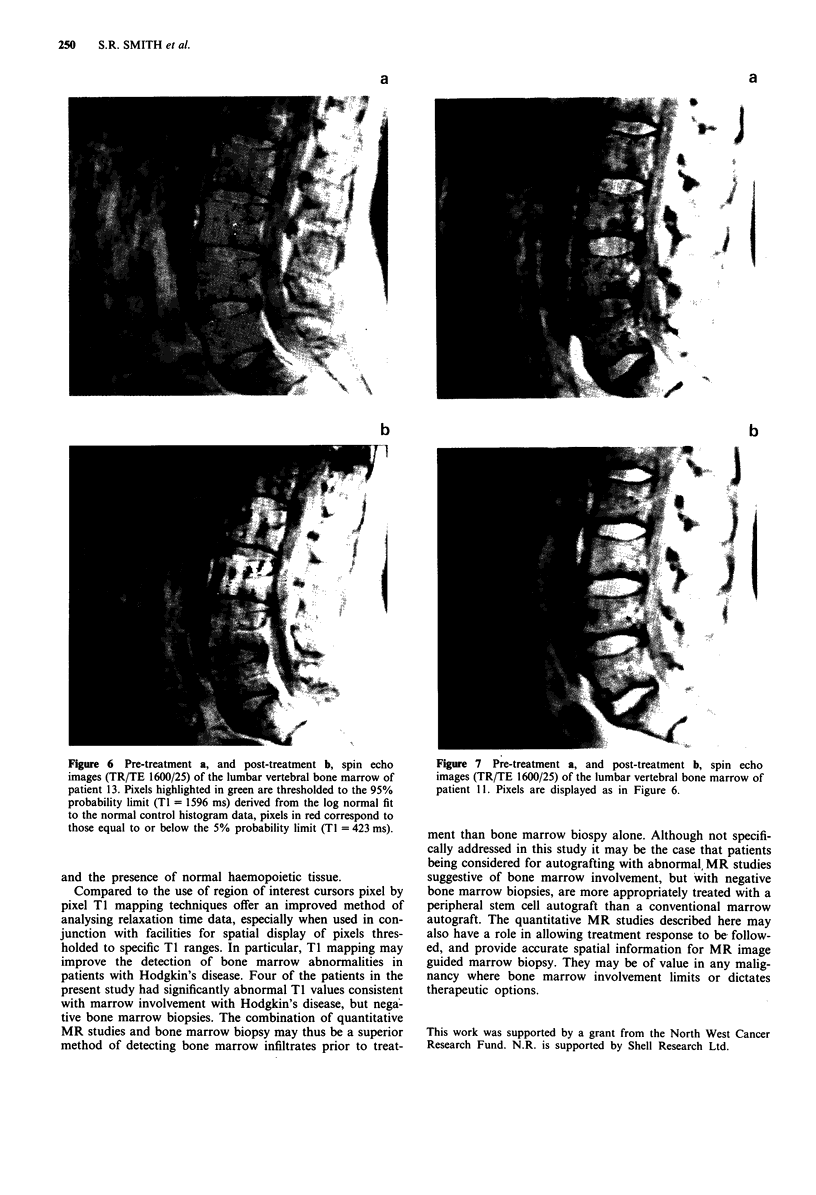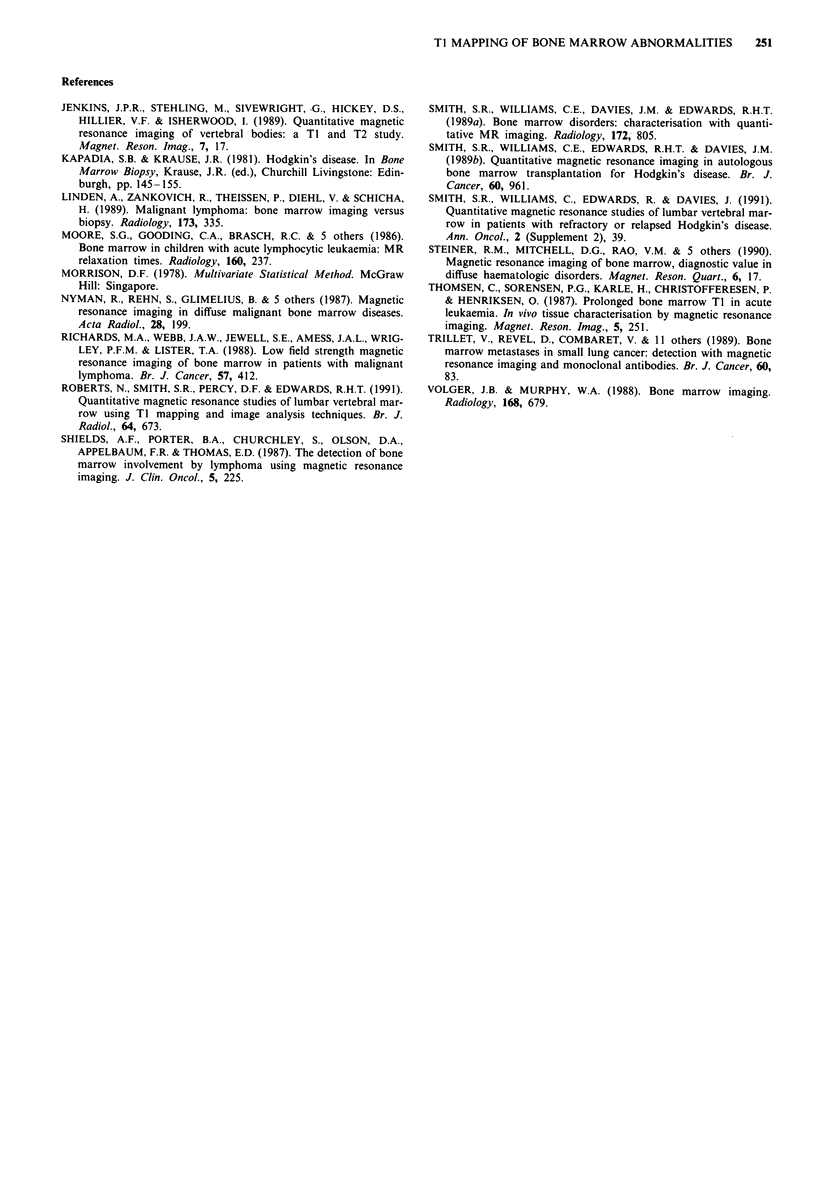# Detection of bone marrow abnormalities in patients with Hodgkin's disease by T1 mapping of MR images of lumbar vertebral bone marrow.

**DOI:** 10.1038/bjc.1992.49

**Published:** 1992-02

**Authors:** S. R. Smith, N. Roberts, D. F. Percy, R. H. Edwards

**Affiliations:** Magnetic Resonance Research Centre, University of Liverpool, UK.

## Abstract

**Images:**


Br. J. Cancer (1992), 65, 246-251 © Macmillan Press Ltd., 1992

### Detection of bone marrow abnormalities in patients with Hodgkin's disease by T1 mapping of MR images of lumbar vertebral bone marrow

S.R. Smith[1,2], N. Roberts[1], D.F. Percy[3] & R.H.T. Edwards[1]

[1]*Magnetic Resonance Research Centre, and Departments of* [2]*Haematology, and* [3]*Statistics and Computational Mathematics, University of Liverpool, PO Box 147, Liverpool, L69 3BX, UK.*

**Summary** Pixel by pixel T1 mapping of MR images has been used in conjunction with image analysis techniques to study the lumbar vertebral bone marrow of 20 patients with refractory or relapsed Hodgkin's disease prior to salvage chemotherapy, or high dose chemotherapy with autologous bone marrow rescue. Compared to 18 age matched controls, seven patients had significantly abnormal lumbar vertebral marrow T1 histograms with median, 5% and 95% centile T1 values that lay outside a three dimensional 95% probability region obtained for the normals. Six of these patients had increased areas of high T1 consistent with bone marrow involvement with lymphoma; although, only in two of them had Hodgkin's disease been detected by bilateral iliac crest bone marrow biopsy. Post-treatment studies were performed in four patients who had abnormal areas of high T1 in the lumbar vertebral bone marrow. All showed normalisation of the T1 histogram, with a reduction of T1 values to within the normal range as defined by the studies of age matched controls.

T1 mapping of bone marrow offers potential for detecting bone marrow infiltrates in patients with lymphoma, and also for the assessment of treatment response.

Bone marrow involvement by lymphoma is conventionally detected by bone marrow biopsy from the posterior iliac crest. This invasive procedure removes a small core of marrow and is undoubtedly subject to sampling errors, particularly in disorders such as Hodgkin's disease that may affect the marrow focally (Kapadia & Krause, 1981). Accurate documentation of marrow involvement by lymphoma is, however, important in establishing the correct stage of disease at initial presentation, and also for assessing disease state prior to procedures such as autologous bone marrow transplantation. A means of improving the detection of marrow infiltrates would therefore have important therapeutic implications.

Magnetic Resonance Imaging, MRI, allows relatively large volumes of marrow to be examined non-invasively and the role of MRI in bone marrow imaging has recently been reviewed (Vogler & Murphy, 1988; Steiner *et al.*, 1990). Compared to bone marrow scintigraphy MRI is more sensitive to changes in marrow fat, gives superior anatomic detail (Steiner *et al.*, 1990), and is also superior to scintigraphy in detecting marrow infiltrates (Shields *et al.*, 1987; Linden *et al.*, 1989).

Proton relaxation times (T1 and T2) can be measured from MR images and provide a quantitative approach to characterising tissues. An elevated thoraco-lumbar vertebral marrow T1 relaxation time is a sensitive indicator of marrow infiltration in patients with lymphoma (Richards *et al.*, 1988), and the observation of changes in T1 after chemotherapy provides a method of objectively assessing treatment response in bone marrow disorders (Moore *et al.*, 1986; Thomsen *et al.*, 1987; Smith *et al.*, 1989*b*). In addition, studies using region of interest cursors have revealed that an increased variation in T1 throughout the lumbar vertebral bone marrow may indicate underlying pathology, by reflecting focal marrow infiltration and marrow heterogeneity (Smith *et al.*, 1991). Relaxation time measurements obtained with region of interest cursors are however subject to limitations, especially when the tissues being examined are known to be heterogeneous (Jenkins *et al.*, 1989).

In the study to be reported here pixel by pixel T1 mapping has been used in conjunction with image analysis techniques (Roberts *et al.*, 1991), to study the lumbar vertebral bone marrow of patients with refractory or relapsed Hodgkin's disease. The aims of the study were to evaluate T1 mapping procedures as a means of detecting bone marrow abnormalities, or infiltration, in patients with lymphoma, and to use a combination of image and statistical analysis techniques to assess bone marrow disease before and after chemotherapy.

### Materials and methods

### *Patients and normal volunteers*

Twenty patients (nine females, age range 19–46 years) with refractory or relapsed Hodgkin's disease underwent quantitative MR studies of the lumbar vertebral bone marrow as part of their assessment prior to autologous bone marrow transplantation, ABMT ($n = 19$), or prior to commencing salvage chemotherapy ($n = 1$). All patients had bilateral posterior iliac crest bone marrow biopsies performed 1 to 3 weeks before the MR examination, no treatment being given in the intervening period.

Nineteen patients had failed two modalities of treatment; either two different chemotherapeutic regimes or a combination of chemo- and radio-therapy schedules (one patient, i.e. number 18, had received fractionated radiotherapy to the lumbar spine). Patient number 13 had relapsed 14 months after attaining a remission with conventional chemotherapy and was being restaged prior to commencing salvage chemotherapy. Patient characteristics and sites of previous radiotherapy are summarised in Table I.

Bone marrow involvement with Hodgkin's disease had been detected by bone marrow biopsy in patients 9 and 12. These two patients received further debulking cyclical chemotherapy with harvesting and cryopreservation of autologous peripheral stem cells, and then received intensive high dose chemotherapy with autologous peripheral stem cell rescue. The other 18 patients had no evidence of bone marrow infiltration on bilateral iliac crest bone marrow biopsy; 17 received high dose chemotherapy with autologous marrow rescue, and one (i.e. patient 13) received conventional dose salvage chemotherapy.

Follow up MRI studies were performed in four of six patients for whom increased T1 values for the vertebral

Correspondence: S.R. Smith, Department of Haematology, Freeman Hospital, Freeman Road, Newcastle upon Tyne, UK.
Received 5 June 1991; and in revised form 15 October 1991.

**Table I** T1 histogram analyses of lumbar vertebral marrow of patients with Hodgkin's disease

| Patient no. | Age | Sex | Previous radiotherapy | T1 value (ms) 5% Centile | Median | 95% Centile | Test value[a] |
|---|---|---|---|---|---|---|---|
| 1 | 35 | M | – | 380 | 620 | 1180 | 1.81 |
| 2 | 33 | M | – | 400 | 740 | 2120 | 6.85 |
| 3 | 37 | F | – | 200 | 280 | 440 | 13.22 |
| 4 | 46 | F | Inverted Y | 320 | 540 | 780 | 5.94 |
| 5 | 25 | F | Mantle | 620 | 940 | 1600 | 2.35 |
| 6 | 41 | F | – | 420 | 600 | 1080 | 3.12 |
| 7 | 44 | M | – | 540 | 860 | 1520 | 0.45 |
| 8 | 44 | M | Mantle | 480 | 820 | 1340 | 1.96 |
| 9 | 45 | M | Mantle | 560 | 1460 | 3960 | 71.70 |
| 10 | 35 | F | – | 320 | 900 | 4180 | 88.48 |
| 11 | 38 | M | – | 440 | 720 | 2300 | 14.16 |
| 12 | 23 | M | Mantle | 600 | 1600 | 3880 | 92.37 |
| 13 | 32 | M | – | 720 | 1600 | 4280 | 61.99 |
| 14 | 32 | F | Mantle | 320 | 460 | 740 | 6.21 |
| 15 | 28 | F | – | 460 | 700 | 1220 | 1.11 |
| 16 | 29 | F | Mantle | 460 | 680 | 1440 | 2.40 |
| 17 | 22 | F | Local neck | 320 | 500 | 760 | 5.49 |
| 18 | 19 | M | Lumbar spine | 200 | 340 | 700 | 10.78 |
| 19 | 24 | M | – | 460 | 640 | 1080 | 3.45 |
| 20 | 26 | M | Mantle | 300 | 720 | 2480 | 20.37 |

[a]Significance level of $P<0.05$ reached when test value $>11.8$.

marrow were observed in the pretreatment MRI studies. Patients 10, 11 and 12 were studied between 10 and 15 weeks post ABMT after peripheral blood counts had normalised, and patient 13 was studied for the second time after three cycles of salvage chemotherapy. Patients 9 and 20 declined further follow up investigation.

Eighteen normal volunteers (11 females, age range 20–45 years) had quantitative MR studies of the lumbar vertebral marrow performed and formed an age matched control group.

*MR studies*

MR studies were performed on a 1.5 Tesla SIGNA system (General Electric company, Milwaukee, USA). The T1 data set consists of six sagittal images of a 10 mm thick midline slice of the lumbar vertebral bodies with repetition times varying from 2400 to 250 ms and a fixed echo time of 25 ms. The details of the imaging protocol have been described elsewhere (Smith *et al.*, 1989*a*). The field of view is 24 cm with an acquisition matrix of 128 × 256 interpolated for display as a square array of pixels each of size 0.94 $mm^2$. The acquisition of each T1 data set took approximately 40 min.

*Data analysis*

Pixel by pixel T1 maps were computed from the T1 data sets of the normal volunteers and patients on a SUN 3/160 workstation (SUN Microsystems, California, USA) as in Roberts *et al.* (1991); the computation took several hours to complete, however, on the latest generation of workstations this could be reduced to less than an hour. Subsequently, using line detection algorithms available on a GOP 302 image analysis system (Struers Vision AB, Sweden), masks were produced that enabled the pixels containing relaxation time data of the bone marrow of the lumbar vertebral bodies to be isolated objectively. Histograms of these T1 data contain the values from several thousand pixels, and analysis of them has been performed as a development of the methods described in Roberts *et al.* (1991).

From the T1 histograms of the normal volunteers median, 5% and 95% centile values were determined and analysed using multivariate normal distribution theory (Morrison, 1978) programmed in the statistical software MINITAB (Minitab Inc., Pennsylvania, USA). A mean vector and covariance matrix were determined, and a series of probability ellipsoids derived (see left hand side of equation 1 below) containing those values appropriate for the specified probability level. In particular, the test value of 11.8 on the right hand side of equation 1 defines the threshold limit for the ellipsoid of 95% probability for the normal volunteers

$$ax_1^2 + bx_2^2 + cx_3^2 + dx_1x_2 + ex_1x_3 + fx_2x_3 < 11.8 \quad (1)$$

where; $x_1$ (5% centile) = 497.2; $x_2$ (median) = 814.4; $x_3$ (95% centile) = 1586.7; a = 0.000689; b = 0.000425; c = 0.0000178; d = − 0.0009541; e = 0.0000957; f = − 0.000120.

Next, values for the median, 5% and 95% centiles obtained from the T1 histograms of the patients with Hodgkin's disease were in turn substituted in the left hand side of equation 1. If the test value was $>11.8$ then the test result was significantly different from those obtained for the controls at the $P = 0.05$, i.e. 95%, probability level.

To assess the relative importance of the median, 5% and 95% centile histogram variables, paried analyses of the above variables were also performed and tested for their ability to discriminate patients from controls; i.e. 5% and median, 5% and 95%, and median and 95%; here the 95% probability region forms an ellipse rather than the three-dimensional ellipsoid of the three variable study.

The above multi-variate analysis procedures enable patients with anomalous values of the 5%, 50% or 95% percentile values to be identified. In a second, and independent, analysis procedure the spatial distribution of the pixels within the 5% and 95% centile was assessed. The lumbar vertebral bone marrow T1 histogram data of the volunteers were noted to exhibit a log normal distribution, and from a log normal fit to the pooled data, one tailed 5% and 95% T1 probability limits (PL) were derived. Using these probability limits as threshold values (lower 5% PL T1 = 423 ms, upper 95% PL T1 = 1596 ms), the percentages of 'low' and 'high' pixels could be calculated for each of the patients studied, and their spatial distribution investigated by means of colour overlays on original grey scale images (Roberts *et al.*, 1991).

Treatment response in the patients studied serially was assessed by both of the methods described above. Firstly, by analysis of differences in the post- as compared to the pre-treatment T1 histogram and, secondly, by monitoring changes in the area and spatial distribution of pixels thresholded to the 95% probability limit of the pooled normal data.

### Results

*Normal volunteers*

Histogram analyses were performed on 18 normal volunteers. A typical T1 histogram of a normal volunteer is shown in Figure 1. The ranges of the 5% and 95% centile T1 values derived from the histograms of normal lumbar vertebral bone marrow were 360 to 660 ms, and 960 and 2340 ms respectively. The median values ranged from 560 to 1060 ms.

*Patient studies*

The median, 5% and 95% centile T1 values derived from the histograms of the 20 patients with Hodgkin's disease are shown in Table I, together with the test values calculated from the quadratic form described by equation 1. Seven patients had abnormal histograms compared with controls as defined by the median, 5% and 95% centile T1 values; i.e. the test values derived from the left hand side of equation (1) were greater than 11.8, and therefore significant at the $P = 0.05$ level.

Six of the above seven patients (i.e. patients 9 to 13, and 20) had lumbar vertebral marrow histograms with abnormally high T1 values, and this finding is consistent with bone marrow infiltration in Hodgkin's disease. The other patient with an elevated test value (patient 3) had a lumbar vertebral marrow T1 histogram with abnormally low median, 5% and 95% centile T1 values. For patients 9 and 12 bone marrow infiltration with Hodgkin's disease had been recognised by

**Figure 1** T1 histogram of lumbar vertebral marrow of normal 28 year old male volunteer.

**Figure 2** Scatter plot showing median and 95% centile T1 histogram values of the 20 patients with Hodgkin's disease (▲). A 95% probability region calculated from the median and 95% centile T1 values of normal age matched controls is shown.

**Figure 3** Pre-treatment **a**, and post-treatment **b**, T1 histograms of lumbar vertebral marrow of patient 13 with Hodgkin's disease.

bone marrow biospy, and both had abnormal T1 histograms with a shift to high T1 values. The other four patients with right shifted T1 histograms had no evidence of marrow infiltration on biopsy.

The analysis of combinations of pairs of histogram variables showed that the median and 95% centile T1 values identified the same seven patients with abnormal histograms as did the trivariate analysis. Patient 18 was also noted to have significantly low T1 values using this paired analysis. A scatter plot of the median and 95% centile data of the 20 patients studied, and the 95% probability ellipse defined from the same two variables for the controls is shown in Figure 2. Examples of the abnormal T1 histograms of the lumbar vertebral marrow obtained for two of the patients are shown in Figures 3a and 4a.

### *Serial studies*

Four patients with abnormal pre-treatment T1 studies had repeat MR examinations following either intensive chemotherapy with autologous bone marrow rescue ($n = 3$) or salvage chemotherapy ($n = 1$). Following treatment all four patients showed normalisation of T1 histograms (Figure 5). The effects of treatment on values obtained from the T1 histogram and the percentage areas of high T1 pixels, following thresholding to the 95% probability limit (i.e. T1 = 1596 ms), are summarised in Table II. The post-treatment histograms of patients 11 and 13 are shown in Figure 3b and 4b, and the effects of treatment on the number and spatial distribution of pixels with T1 values greater than the 95% probability limit are shown in Figures 6 and 7.

### Discussion

The aim of this study was to assess the ability of T1 mapping procedures to document bone marrow involvement in patients with Hodgkin's disease. Conventional spin echo MR imaging has been shown to be of value in detecting marrow infiltration in lymphoma and small cell lung cancer, but this relies on the subjective interpretation of often subtle alterations in signal intensity patterns (Linden *et al.*, 1989; Trillet *et al.*, 1989). Quantitative approaches measuring marrow relaxation times using region of interest cursors show promise (Richards *et al.*, 1988; Smith *et al.*, 1989*b*), but suffer from the limitations associated with the use of such cursors (Jenkins *et al.*, 1989). The T1 mapping procedures used in the present study offer several advantages over region of interest cursor methods.

Line detection routines requiring minimal operator interaction, were used to objectively isolate T1 relaxation time data from only the lumbar vertebral marrow. All the pixel by pixel T1 data in this region was then displayed in histogram

Figure 4 Pre-treatment a, and post-treatment b, T1 histograms of lumbar vertebral marrow of patient 11 with Hodgkin's disease.

**Figure 5** The effects of treatment on the median and 95% centile T1 histogram values of the four patients with Hodgkin's disease studied serially. The 95% probability region calculated from the median and 95% centile T1 values of controls is also shown.

form and the major moments of the histogram analysed. In addition, the ability to identify pixels with T1 values thresholded to a specific T1 range allowed the spatial distribution of abnormal areas to be studied, and the effect of treatment upon this distribution to be followed in detail. The analyses of histogram data initially used the combination of the median, 5% and 95% centile values for identifying patients with anomolous lumbar vertebral marrow. However, a more straight-forward two dimensional approach, easier to depict graphically and giving similar results, was shown to be possible considering only the median and 95% centile values of the histogram.

The two patients with positive bone marrow biopsies (i.e. patients 9 and 12) had significantly abnormal T1 histograms compared to age matched controls. The histogram analyses gave high median and 95% centile values, and greatly increased areas of T1 pixels thresholded to the 95% probability limit. In addition, four patients with negative bone marrow biopsies had abnormal quantitative MR studies with T1 histograms exhibiting a shift towards high values. Two patterns were noted, either abnormally high median and 95% centile values, or high 95% centile values alone. These right shifted T1 histogram patterns may respectively represent either the diffuse or focal patterns of marrow infiltration seen in Hodgkin's disease.

What do these high T1 pixels in the lumbar marrow represent? The T1 value of bone marrow within a pixel reflects the average of the signal from short T1 fat, and the long T1 of the water in its various water-macromolecular environments in normal, or abnormal, haemopoietic tissue. Studies by Nyman *et al.* (1987), and Smith *et al.* (1989*a*), have shown that the more cellular the bone marrow the longer the T1. The high T1 pixels in the patients with Hodgkin's disease therefore probably represent focal areas of marrow infiltration with Hodgkin's disease, with their associated increase in cellularity and/or fibrous tissue, or possibly areas of hypercellular reactive marrow. Conversely, in patient 18 the observation of significantly low T1 values is consistent with hypocellular lumbar vertebral bone marrow following radiotherapy to the lumbar spine. Patient 3, who also had an unusually large number of low T1 values, had hypocellular bone marrow biopsies following multiple courses of myelosupppressive chemotherapy.

Four patients were studied following treatment and after peripheral blood counts had recovered. In all cases histogram shape, and areas of marrow occupied by high T1 pixels normalised, suggesting a good response to treatment. Reductions in T1 following treatment are consistent with elimination of Hodgkin's disease from the marrow and replacement with normocellular haemopoietic tissue. This is supported by the fact that repeat bone marrow biopsies in patient 12 showed elimination of Hodgkin's disease from the marrow

**Table II** Effects of treatment on histogram discriptors

| | *Pre-treatment* | | | *Post-treatment* | | |
|---|---|---|---|---|---|---|
| *Patient* | *Median* | *95% Centile* | *Area occupied by pixels thresholded to T1 of 1596 ms* | *Median* | *95% Centile* | *Area occupied by pixels thresholded to T1 of 1596 ms* |
| 10 | 900 | 4180 | 28.8 | 400 | 860 | 1.05 |
| 11 | 720 | 2300 | 12.9 | 660 | 1140 | 0.95 |
| 12 | 1600 | 3880 | 50.1 | 560 | 960 | 0.2 |
| 13 | 1600 | 4280 | 49.7 | 600 | 1220 | 2.5 |

The threshold value of 1596 ms corresponds to the 95% probability limit derived from log normal fit to normal control data. Median and 95% T1 values are expressed in milliseconds. Area is expressed as a percentage of total pixels sampled.

**Figure 6** Pre-treatment **a**, and post-treatment **b**, spin echo images (TR/TE 1600/25) of the lumbar vertebral bone marrow of patient 13. Pixels highlighted in green are thresholded to the 95% probability limit (T1 = 1596 ms) derived from the log normal fit to the normal control histogram data, pixels in red correspond to those equal to or below the 5% probability limit (T1 = 423 ms).

**Figure 7** Pre-treatment **a**, and post-treatment **b**, spin echo images (TR/TE 1600/25) of the lumbar vertebral bone marrow of patient 11. Pixels are displayed as in Figure 6.

and the presence of normal haemopoietic tissue.

Compared to the use of region of interest cursors pixel by pixel T1 mapping techniques offer an improved method of analysing relaxation time data, especially when used in conjunction with facilities for spatial display of pixels thresholded to specific T1 ranges. In particular, T1 mapping may improve the detection of bone marrow abnormalities in patients with Hodgkin's disease. Four of the patients in the present study had significantly abnormal T1 values consistent with marrow involvement with Hodgkin's disease, but negative bone marrow biopsies. The combination of quantitative MR studies and bone marrow biopsy may thus be a superior method of detecting bone marrow infiltrates prior to treatment than bone marrow biospy alone. Although not specifically addressed in this study it may be the case that patients being considered for autografting with abnormal, MR studies suggestive of bone marrow involvement, but with negative bone marrow biopsies, are more appropriately treated with a peripheral stem cell autograft than a conventional marrow autograft. The quantitative MR studies described here may also have a role in allowing treatment response to be followed, and provide accurate spatial information for MR image guided marrow biopsy. They may be of value in any malignancy where bone marrow involvement limits or dictates therapeutic options.

This work was supported by a grant from the North West Cancer Research Fund. N.R. is supported by Shell Research Ltd.

### References

JENKINS, J.P.R., STEHLING, M., SIVEWRIGHT, G., HICKEY, D.S., HILLIER, V.F. & ISHERWOOD, I. (1989). Quantitative magnetic resonance imaging of vertebral bodies: a T1 and T2 study. *Magnet. Reson. Imag.*, **7**, 17.

KAPADIA, S.B. & KRAUSE, J.R. (1981). Hodgkin's disease. In *Bone Marrow Biopsy*, Krause, J.R. (ed.), Churchill Livingstone: Edinburgh, pp. 145–155.

LINDEN, A., ZANKOVICH, R., THEISSEN, P., DIEHL, V. & SCHICHA, H. (1989). Malignant lymphoma: bone marrow imaging versus biopsy. *Radiology*, **173**, 335.

MOORE, S.G., GOODING, C.A., BRASCH, R.C. & 5 others (1986). Bone marrow in children with acute lymphocytic leukaemia: MR relaxation times. *Radiology*, **160**, 237.

MORRISON, D.F. (1978). *Multivariate Statistical Method*. McGraw Hill: Singapore.

NYMAN, R., REHN, S., GLIMELIUS, B. & 5 others (1987). Magnetic resonance imaging in diffuse malignant bone marrow diseases. *Acta Radiol.*, **28**, 199.

RICHARDS, M.A., WEBB, J.A.W., JEWELL, S.E., AMESS, J.A.L., WRIGLEY, P.F.M. & LISTER, T.A. (1988). Low field strength magnetic resonance imaging of bone marrow in patients with malignant lymphoma. *Br. J. Cancer*, **57**, 412.

ROBERTS, N., SMITH, S.R., PERCY, D.F. & EDWARDS, R.H.T. (1991). Quantitative magnetic resonance studies of lumbar vertebral marrow using T1 mapping and image analysis techniques. *Br. J. Radiol.*, **64**, 673.

SHIELDS, A.F., PORTER, B.A., CHURCHLEY, S., OLSON, D.A., APPELBAUM, F.R. & THOMAS, E.D. (1987). The detection of bone marrow involvement by lymphoma using magnetic resonance imaging. *J. Clin. Oncol.*, **5**, 225.

SMITH, S.R., WILLIAMS, C.E., DAVIES, J.M. & EDWARDS, R.H.T. (1989*a*). Bone marrow disorders: characterisation with quantitative MR imaging. *Radiology*, **172**, 805.

SMITH, S.R., WILLIAMS, C.E., EDWARDS, R.H.T. & DAVIES, J.M. (1989*b*). Quantitative magnetic resonance imaging in autologous bone marrow transplantation for Hodgkin's disease. *Br. J. Cancer*, **60**, 961.

SMITH, S.R., WILLIAMS, C., EDWARDS, R. & DAVIES, J. (1991). Quantitative magnetic resonance studies of lumbar vertebral marrow in patients with refractory or relapsed Hodgkin's disease. *Ann. Oncol.*, **2** (Supplement 2), 39.

STEINER, R.M., MITCHELL, D.G., RAO, V.M. & 5 others (1990). Magnetic resonance imaging of bone marrow, diagnostic value in diffuse haematologic disorders. *Magnet. Reson. Quart.*, **6**, 17.

THOMSEN, C., SORENSEN, P.G., KARLE, H., CHRISTOFFERESEN, P. & HENRIKSEN, O. (1987). Prolonged bone marrow T1 in acute leukaemia. *In vivo* tissue characterisation by magnetic resonance imaging. *Magnet. Reson. Imag.*, **5**, 251.

TRILLET, V., REVEL, D., COMBARET, V. & 11 others (1989). Bone marrow metastases in small lung cancer: detection with magnetic resonance imaging and monoclonal antibodies. *Br. J. Cancer*, **60**, 83.

VOLGER, J.B. & MURPHY, W.A. (1988). Bone marrow imaging. *Radiology*, **168**, 679.